# Comparative Evaluation of Chest Ultrasonography and Computed Tomography as Predictors of Malignant Pleural Effusion: A Prospective Study

**DOI:** 10.3390/diagnostics14101041

**Published:** 2024-05-17

**Authors:** Samah M. Shehata, Yassir Edrees Almalki, Mohammad Abd Alkhalik Basha, Rasha Mohamed Hendy, Eman M. Mahmoud, Marwa Elsayed Abd Elhamed, Sharifa Khalid Alduraibi, Mervat Aboualkheir, Ziyad A. Almushayti, Alaa K. Alduraibi, Ahmed M. Abdelkhalik Basha, Maha E. Alsadik

**Affiliations:** 1 Department of Chest Disease, Faculty of Human Medicine, Zagazig University, Zagazig 44519, Egypt; sama7she7ata78@gmail.com (S.M.S.); mahaalsadik@gmail.com (M.E.A.); 2 Division of Radiology, Department of Internal Medicine, Medical College, Najran University, Najran 61441, Saudi Arabia; 3 Department of Diagnostic Radiology, Faculty of Human Medicine, Zagazig University, Zagazig 44519, Egypt; mohammad_basha76@yahoo.com (M.A.A.B.); dr.meroelsayed34@gmail.com (M.E.A.E.); 4 Department of Chest Disease, Faculty of Human Medicine, Benha University, Benha 13511, Egypt; rashahendy97@yahoo.com; 5 Department of Chest Disease, Faculty of Human Medicine, Port Said University, Port Said 42511, Egypt; emanmahmoud597@gmail.com; 6 Department of Radiology, College of Medicine, Qassim University, Buraidah 52571, Saudi Arabia; salduraibi@qu.edu.sa (S.K.A.); ziyadalmushayti@qu.edu.sa (Z.A.A.); al.alderaibi@qu.edu.sa (A.K.A.); 7 Department of Internal Medicine, College of Medicine, Taibah University, Madinah 42353, Saudi Arabia; maboualkheir@taibahu.edu.sa; 8 Faculty of General Medicine, Saint Petersburg State University, Egypt Branch, Cairo 11646, Egypt; ahm7edbasha@gmail.com

**Keywords:** malignant pleural effusion, chest ultrasonography, chest computed tomography, diagnostic accuracy, biopsy

## Abstract

Malignant pleural effusion (MPE) is a manifestation of advanced cancer that requires a prompt and accurate diagnosis. Ultrasonography (US) and computed tomography (CT) are valuable imaging techniques for evaluating pleural effusions; however, their relative predictive ability for a malignant origin remains debatable. This prospective study aimed to compare chest US with CT findings as predictors of malignancy in patients with undiagnosed exudative pleural effusion. Fifty-four adults with undiagnosed exudative pleural effusions underwent comprehensive clinical evaluation including chest US, CT, and histopathologic biopsy. Blinded radiologists evaluated the US and CT images for features suggestive of malignancy, based on predefined criteria. Diagnostic performance measures were calculated using histopathology as a reference standard. Of the 54 patients, 33 (61.1%) had MPEs confirmed on biopsy. No significant differences between US and CT were found in detecting parietal pleural abnormalities, lung lesions, chest wall invasion, or liver metastasis. US outperformed CT in identifying diaphragmatic pleural thickening ≥10 mm (33.3% vs. 6.1%, *p* < 0.001) and nodularity (45.5% vs. 3%, *p* < 0.001), whereas CT was superior for mediastinal thickening (48.5% vs. 15.2%, *p* = 0.002). For diagnosing MPE, diaphragmatic nodularity detected by US had 45.5% sensitivity and 100% specificity, whereas CT mediastinal thickening had 48.5% sensitivity and 90.5% specificity. Both US and CT demonstrate reasonable diagnostic performance for detecting MPE, with particular imaging findings favoring a malignant origin. US may be advantageous for evaluating diaphragmatic pleural involvement, whereas CT is more sensitive to mediastinal abnormalities.

## 1. Introduction

Malignant pleural effusion (MPE) is a particular form of pleural effusion commonly associated with advanced-stage malignancies, such as lung cancer, breast cancer, lymphoma, and metastatic diseases from other primary tumors [1]. Early and accurate diagnosis of MPE is crucial to initiate appropriate treatment and determine prognosis [2,3,4]. Traditionally, the diagnostic process for suspected MPE involves thoracentesis, which includes cytological and biochemical analyses. However, this invasive procedure poses risks, such as pneumothorax, bleeding, and patient discomfort. Moreover, the diagnostic yield of thoracentesis can be limited, with false-negative rates ranging from 20% to 40% due to factors like paucicellular effusions or suboptimal sampling techniques [4,5,6]. In recent years, noninvasive imaging modalities such as ultrasonography (US) and computed tomography (CT) have become increasingly important in evaluating pleural effusions and diagnosing MPE. These modalities can provide valuable information about the nature and characteristics of pleural effusion, potentially guiding clinical decision making and reducing the need for invasive procedures [7,8,9].

Chest US and CT are two commonly used imaging modalities for evaluating pleural effusion. While both methods provide valuable information, they have their advantages and limitations [10,11]. Chest US is a widely available and cost-effective imaging technique that has been increasingly used for assessing pleural effusion. It offers benefits such as portability, the absence of ionizing radiation, and the ability to accurately guide procedures, like thoracentesis. US allows real-time visualization of the pleural space, facilitating the identification and evaluation of pleural effusions, including their characteristics (e.g., echogenicity, septations, and loculations). It also assists in guiding thoracentesis and other interventional procedures. Additionally, chest US can aid in the detection of pleural nodules, thickening, and other abnormalities suggestive of malignancy [12,13,14]. On the other hand, chest CT has long been recognized as a valuable diagnostic tool in evaluating thoracic pathologies, including pleural effusions. CT scans provide detailed anatomical information, enabling the assessment of pleural effusion characteristics such as size, complexity, and associated pleural or parenchymal abnormalities [15,16]. Furthermore, CT can aid in identifying underlying malignancies or metastatic lesions that may contribute to pleural effusion. CT scans are particularly useful when the etiology of the effusion is unclear or when loculated or complex effusions are suspected [16,17]. However, the widespread availability and accessibility of CT may be limited in certain healthcare settings, and it involves exposure to ionizing radiation and contrast agents, which may not be ideal for some patients, especially those with renal impairment or allergies [18].

The choice between chest US and chest CT as predictors of MPE depends on several factors, such as the clinical context, resource availability, and the specific information needed for patient management [7,19]. Although both US and CT have been extensively studied for their diagnostic utility in evaluating pleural effusions, there is still ongoing debate regarding their relative predictive value in determining the malignant nature of the effusions. Some studies have suggested that chest US may be as accurate as CT in distinguishing malignant from benign pleural effusions, while others have favored CT as a more reliable predictor [20,21,22,23,24]. The present study aims to compare chest US and chest CT as predictors of malignant features in undiagnosed exudative pleural effusions, using histopathologic biopsy as the reference standard for determining the malignant or benign nature of the effusion. By critically analyzing the strengths and limitations of each imaging modality, this study seeks to provide valuable insights into the optimal utilization of chest US and CT in the diagnostic workup of pleural effusions, particularly in cases where there is clinical suspicion of malignancy. These findings may assist clinicians in selecting the most appropriate imaging modality or using a combination of techniques to improve diagnostic accuracy and optimize patient management.

## 2. Methods

### 2.1. Ethical Statement

This study received approval from the Institutional Review Board (approval number ZU-10057) on 13 November 2022. All participants provided informed consent before participating. This study was conducted in accordance with the ethical principles of the Declaration of Helsinki.

### 2.2. Study Design and Eligibility Criteria

This prospective study involved 54 patients who had previously undiagnosed exudative pleural effusion. This study took place at the chest department of the Zagazig University Hospital between November 2022 and May 2023. This study followed specific inclusion and exclusion criteria. The inclusion criteria were as follows: (i) patients aged ≥ 18 years, (ii) evidence of pleural effusion observed on chest X-ray, (iii) presence of exudative pleural effusion, and (iv) no previous diagnosis of the etiology of pleural effusion. Exclusion criteria included: (i) patients with a previous clinical and/or histopathological diagnosis of pleural effusion, (ii) patients with a transudative pleural effusion, (iii) patients with a bleeding tendency (e.g., INR > 1.7, prothrombin activity < 40%, platelet count < 40,000/mL), or (iv) pregnant patients. The following examinations were conducted on all the enrolled patients: (i) detailed medical history and physical examination; (ii) laboratory tests, including complete blood count, renal function tests, and coagulation profile (PT, PTT, INR); (iii) comprehensive pleural fluid analysis (including protein, LDH, glucose, pH, adenosine deaminase, total and differential white blood cell counts, microbiological cultures, including mycobacterium tuberculosis, and cytologic analysis); and (iv) chest imaging, which included chest radiography, US, and CT scan.

### 2.3. Chest US Protocol

All chest US examinations were performed using the Sonoscape SSI 4000 US system (Sonoscape Co. Ltd., Shenzhen, China). A consultant radiologist (M.E.A.E.) with 8 years of experience in chest imaging comprehensive chest US examinations. The radiologist was blinded to the clinical details. A 3.5 MHz convex array probe was used for pleura and lung scanning, while a 10 MHz linear array transducer was used to visualize the parietal pleura and chest wall details. The patient was positioned sitting with arms crossed, although the lateral decubitus position was used for some patients. Both hemithoraces were systematically scanned, moving the transducer from the posterior to the anterior of the chest along the intercostal spaces and from the diaphragm to the apex along the chest wall vertical lines to identify pathological features and anatomical landmarks [25]. A sonographic assessment was performed to determine the presence, size, complexity, and distribution of pleural effusion. The pleural line was thoroughly examined to detect any abnormalities such as nodularity, thickening (with measurement), or irregularity. The lung parenchyma was closely inspected for subpleural consolidation, nodules, or masses. Color Doppler was used to evaluate the abnormal vascularity of the pleural lesions. Thoracic structures such as the diaphragm, liver, spleen, and heart were surveyed. The sonographic characteristics and patterns suggestive of a benign or malignant etiology of the effusion were documented. To predict pleural malignancy, chest US criteria with a high probability [22] were applied. These criteria were considered met if one or more of the following findings were present: (i) diaphragmatic or pleural nodularity, (ii) diaphragmatic or pleural thickening greater than 10 mm, (iii) an adjacent solid lung lesion, or (iv) liver metastases. The presence of any of these sonographic features was highly suggestive of MPE etiology.

### 2.4. Chest CT Protocol

All patients underwent chest CT scans within 48 h of chest US examination using a 128-multidetector CT scanner (Philips Ingenuity 128, Philips Healthcare, Amsterdam, The Netherlands). The entire thorax, from the lung apices to the level of the adrenal glands, was imaged with the patient in the supine position and instructed to hold a full inspiration. Thin 1 mm slices were obtained at 1 mm intervals using the following settings: 120 kVp, 140 mAs, 0.5 s rotation time, 0.625 mm collimation, 1.1 pitch, 350–500 FOV, and a 512 × 512 matrix. A nonionic low-osmolar intravenous contrast agent (Ultravist 370; Bayer Schering Pharma AG, Berlin, Germany) was administered at a rate of 3–4 mL/sec based on the patient’s weight, with a delay of 60–70 s. The lung window (WW= 1500 HU, WL= −600 HU) and mediastinal window (WW= 400 HU, WL= 40 HU) were used to assess the size, complexity, and presence of pleural effusion, pleural thickening or nodules, lung parenchymal findings, lymphadenopathy, and other thoracic abnormalities. Multiplanar reconstructions (MPR) in the coronal and sagittal planes were also generated and reviewed to optimize the detection of pleural abnormalities, particularly along diaphragmatic surfaces.

### 2.5. CT Image Analysis

All CT images were transferred to the workstation and interpreted using a picture archiving and communication system (PACS) (Paxera Ultima, Paxera Viewer version 5.0.9.6, Paxera Health, Newtone, MA, USA). Another consultant radiologist with more than 13 years of experience in chest imaging (M.A.A.B.) comprehensively analyzed the images. The radiologist was not aware of the clinical data and US results. The analysis included assessing the presence, size, and characteristics (simple/complex, loculated/free-flowing) of pleural effusion. Additionally, pleural abnormalities such as nodularity, thickening (with measurement of maximal thickness), and enhancement were noted. Lung window images were carefully examined for parenchymal abnormalities including consolidation, ground-glass opacities, nodules, and masses. Mediastinal window images were evaluated for signs of lymphadenopathy (a short-axis diameter ≥ 1 cm was considered suspicious). Other thoracic findings, such as masses and bony lesions, were also documented. Patterns of pleural abnormalities suggesting a malignant or benign cause were described. The criteria established by Leung et al. [26] were used to differentiate between malignant and nonmalignant lesions. Finally, the diagnosis obtained from chest US was compared with the definitive diagnosis, as well as the diagnosis obtained from chest CT.

### 2.6. Reference Standard

The final diagnosis of MPE was confirmed by histopathological biopsy findings. Various biopsy procedures were performed, including US-guided biopsy in 11 patients (20.4%), CT-guided biopsy in 16 patients (29.6%), thoracoscopic biopsy in 10 patients (18.5%), bronchoscopic biopsy in four patients (7.4%), and excisional lymph node biopsy in two patients (3.8%). An experienced pathologist blinded to the imaging findings evaluated and reported the pathological results.

### 2.7. Statistical Analysis

Continuous variables were expressed as mean ± SD or median (range), while categorical variables were presented as numbers (percentages). The normality of continuous variables was assessed using the Shapiro–Wilk test. Student’s t-test for independent samples was utilized to compare two groups of normally distributed variables. Percentages of categorical variables were compared using the chi-squared test or Fisher’s exact test, as appropriate. The McNemar test was employed to analyze paired categorical data. Validity was determined by calculating the diagnostic performance based on the construction of 2 × 2 contingency tables. Sensitivity, specificity, positive predictive value (PPV), and negative predictive value (NPV) were calculated. Logistic regression analysis was conducted to evaluate the association between various US and CT findings and the presence of MPE. Adjusted odds ratios (ORs) with corresponding 95% confidence intervals (CIs) were calculated to quantify the strength of the association between each imaging predictor and MPE while controlling for other variables in the model. All tests were two-sided, with a statistical significance level of *p* < 0.05. Statistical analyses were performed using SPSS 22.0 for Windows (SPSS Inc., Chicago, IL, USA) and MedCalc version 13 for Windows (MedCalc Software bvba, Ostend, Belgium).

## 3. Results

### 3.1. Patient Characteristics and Final Diagnoses

Table 1 provides an overview of patient data in the study cohort. A total of 54 patients (63% men) took part in this study. The average age of the patients was 50.8 ± 8.5 years. Thirty-three (61.1%) patients were diagnosed with MPE, while twenty-one (38.9%) had benign effusions. In the patients with benign effusions, the underlying causes were nonspecific inflammatory lesions in 3 patients (5.6%), tuberculosis in 11 patients (20.4%), and pneumonia in 7 patients (13%). The most common cause in the MPE group was metastatic disease, which occurred in 21 (38.8%) patients. Lung cancer was the primary malignancy in 16 patients (29.6%), with adenocarcinoma being the most common histological subtype (11 patients, 20.4%), followed by small-cell lung cancer (5 patients, 9.2%). Other malignancies included breast cancer in 4 patients (7.4%), gastrointestinal cancer in 1 patient (1.9%), and mesothelioma in 12 patients (22.2%). This table illustrates the diverse spectrum of underlying causes contributing to pleural effusions in the study population, emphasizing the importance of accurate diagnostic workup and characterization.

### 3.2. US and CT Findings in MPE

Table 2 shows a comparison of the US and CT findings in 33 patients with MPE. This study highlights the significant differences in detection rates between the two imaging modalities for certain pathological features. In particular, CT was more effective than US in detecting diaphragmatic pleural thickening (≥10 mm), with a prevalence of 33.3% versus 6.1% detected with US (*p* < 0.001). In addition, CT showed a superior ability to detect diaphragmatic pleural nodules (45.5%) compared with US (3%) (*p* < 0.001). Similarly, mediastinal thickening was observed more frequently in CT (48.5%) than in US (15.2%) (*p* = 0.002). In contrast, parietal pleural thickening (≥10 mm) was detected more frequently in US (27.3%) than in CT (39.4%) (*p* = 0.016). Other findings, such as parietal pleural thickening, parietal pleural nodules, pleural rind, peripheral lung lesions, chest wall invasion, and liver metastases, showed no significant differences between the two methods (*p* ≥ 0.05). These findings emphasize the complementary nature of US and CT imaging techniques in the comprehensive evaluation of MPE, with each method offering distinct strengths in detecting specific imaging characteristics.

### 3.3. Imaging Findings in Benign Pleural Effusion

Table 3 shows the US and CT findings of the 21 patients with benign pleural effusion. Most patients showed no signs of malignancy on either procedure. A small group of patients (9.5%, 4.8%, and 4.8% in US and 9.5%, 4.8%, and 9.5% in CT) had parietal pleural thickening, parietal pleural nodule formation, and mediastinal thickening, respectively. Notably, none of the patients showed thickening of the diaphragmatic pleura, diaphragmatic pleural nodules, or pleural rinds in both US and CT. Peripheral lung lesions were observed in 23.8% of patients in US and 28.6% of patients in CT, which could indicate underlying lung pathology or atelectasis. There were no statistically significant differences between US and CT findings in this cohort of benign effusions (*p* > 0.05). These results suggest that the absence of features, such as diaphragmatic involvement, pleural rind, and chest wall invasion may indicate a benign cause. However, the presence of parietal pleural abnormalities may also indicate benign disease. Additionally, the presence of parietal pleural abnormalities or mediastinal thickening does not necessarily imply malignancy.

### 3.4. Validity of US and CT Findings in Detecting MPE

Table 4 shows the validity of various US and CT findings in the detection of MPE compared with histopathology. The most sensitive US findings were diaphragmatic pleural nodules (45.5%) and thickening (42.4%). Parietal pleural and mediastinal thickening showed the highest sensitivity (48.5%) in CT. Both US and CT showed 100% specificity for diaphragmatic pleural thickening, diaphragmatic pleural nodules, pleural rinds, chest wall invasion, and liver metastases. PPVs were generally high, ranging from 85.7% to 100% for most US and CT findings, except for peripheral lung lesions (54.5% for US and 60% for CT). NPVs were relatively low for all imaging findings, with the highest NPV (53.8%) for US-diaphragmatic pleural nodules. These results suggest that while US and CT can accurately identify MPE in the presence of certain findings, their ability to exclude MPE due to missing findings is limited.

### 3.5. Logistic Regression Analysis for MPE

Table 5 presents the results of the logistic regression analysis of MPE using US and CT findings as predictors. Our analysis revealed several significant associations between the imaging findings and MPE. Diaphragmatic pleural nodularity in US was the strongest predictor of MPE (OR = 9.45, 95% CI: 3.56–25.08, *p* < 0.001). Parietal pleural thickening ≥10 mm showed a significant association with MPE on both US (OR = 2.15, 95% CI: 1.02–4.53, *p* = 0.044) and CT (OR = 3.12, 95% CI: 1.28–7.62, *p* = 0.012). Similarly, diaphragmatic pleural thickening ≥10 mm in US was strongly associated with MPE (OR = 5.28, 95% CI: 2.19–12.72, *p* < 0.001). The presence of a pleural rind in CT was also a strong predictor of MPE (OR = 6.82, 95% CI: 2.47–18.86, *p* < 0.001). Mediastinal thickening in CT was also a significant predictor of MPE (OR = 4.67, 95% CI: 1.83–11.92, *p* = 0.001). Liver metastases in US (OR = 3.89, 95% CI: 1.02–14.86, *p* = 0.047) and chest wall invasion in US (OR = 3.25, 95% CI: 0.98–10.76, *p* = 0.054) were also associated with increased odds of MPE, but the confidence intervals were relatively wide, indicating less precise estimates. Several findings, including parietal pleural nodularity in both US and CT, diaphragmatic pleural thickening ≥10 mm and diaphragmatic pleural nodularity in CT, mediastinal thickening in US, and peripheral lung lesions on both modalities and chest wall invasion and liver metastases in CT, were not significantly associated with MPE.

**Figure 1:**
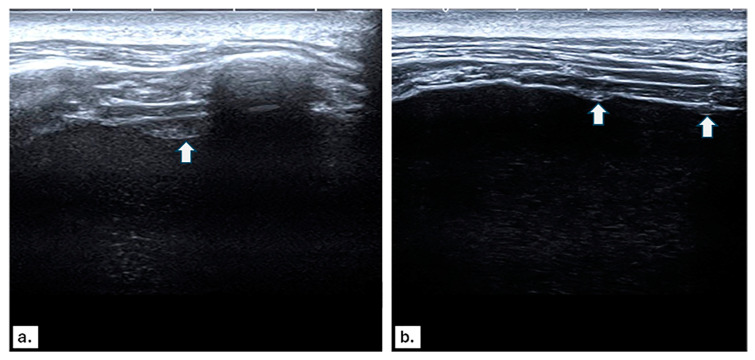
(**a**,**b**) Ultrasound images show massive right-sided pleural effusion with costal pleural nodules (white arrows). US-guided pleural biopsy was performed, and histopathological examination revealed pleural metastases.

**Figure 2:**
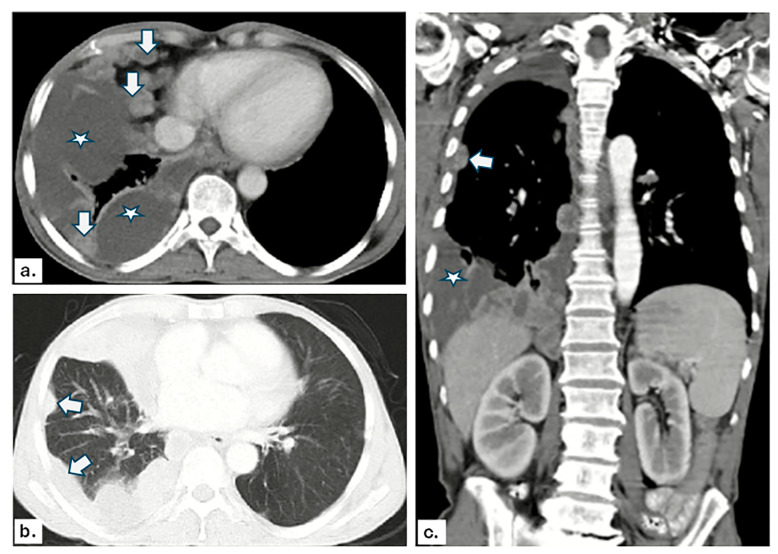
Contrast-enhanced CT: (**a**) axial mediastinal window image, (**b**) axial lung window image, and (**c**) coronal mediastinal window image show right-side pleural effusion (white stars) with multiple heterogeneously enhanced pleural nodules (white arrows). A CT-guided biopsy was performed, and histopathological examination revealed pleural mesothelioma.

Figure 1 and Figure 2 show representative images from our study.

## 4. Discussion

This study aimed to compare the diagnostic performance of chest ultrasonography (US) with computed tomography (CT) in predicting malignant pleural effusion (MPE) using histopathologic biopsy as the gold standard. The results of this study offer valuable insights into the strengths and limitations of each modality in detecting specific imaging features associated with MPE.

CT significantly outperformed US in detecting diaphragmatic pleural thickening (≥10 mm). The detection rate of CT was 33.3%, compared with 6.1% with US (*p* < 0.001). Similarly, CT exhibited a superior ability to identify diaphragmatic pleural nodularity, with a detection rate of 45.5% compared with 3% with US (*p* < 0.001). These findings support previous studies that have highlighted the advantages of CT in visualizing diaphragmatic pleural abnormalities owing to its cross-sectional imaging capabilities and ability to overcome the limitations of air-filled lungs [22,27,28]. However, our findings contradict those of Drawish et al. [29], who found that US was superior to CT in detecting pleural nodules. The higher spatial resolution and lack of air interference with US may explain its better performance in detecting parietal pleural abnormalities compared with diaphragmatic pleural lesions [30].

In contrast to diaphragmatic pleural findings, US demonstrated a higher sensitivity in detecting parietal pleural thickening (≥10 mm) than CT (27.3% vs. 39.4%, *p* = 0.016). This observation aligns with previous studies that have shown the superiority of US in evaluating the parietal pleura because of its better spatial resolution and real-time imaging capabilities [31,32]. However, there was no significant difference between US and CT in the detection of parietal pleural nodularity (30.3% vs. 24.2%, *p* = 0.500). These findings suggest that while US may be more sensitive for detecting parietal pleural thickening, both modalities have comparable performance in identifying parietal pleural nodularity [22].

CT demonstrated a significantly higher detection rate of mediastinal thickening than US (48.5% vs. 15.2%, *p* = 0.002). This result is not surprising because CT is known to offer superior anatomical detail and better visualization of mediastinal structures owing to its cross-sectional imaging capabilities and the ability to overcome limitations imposed by overlying structures [33]. The limited field of view and potential interference from the surrounding structures may explain the lower sensitivity of US in detecting mediastinal abnormalities.

This study discovered that pleural rind was detected exclusively in CT scans (27.3%), while US did not identify any patients with pleural rind. This observation aligns with previous literature, which suggests that CT is more effective in detecting pleural rinds due to its ability to visualize subtle pleural and parenchymal abnormalities [34]. The presence of a pleural rind is considered a specific indicator of malignant pleural disease, and its identification in CT can assist in diagnosing MPE.

There was no significant difference between US and CT in detecting peripheral lung lesions (18.2% vs. 27.3%, *p* = 0.250) or chest wall invasion (12.1% vs. 3%, *p* = 0.250). This finding supports previous literature, that concluded US guidance is comparable to CT guidance for pleural or peripheral lung lesions [35]. While CT may have a slight advantage in detecting these features due to its cross-sectional imaging capabilities and ability to visualize the entire thoracic cavity, US can also provide valuable information, particularly in assessing peripheral lung lesions [30,36].

Both US and CT demonstrated a low detection rate for liver metastasis (9.1% vs. 3%, *p* = 0.500), with no significant difference between the two modalities. While the presence of liver metastasis can be an important finding in the evaluation of MPE, especially in cases of metastatic disease, the relatively low detection rates in this study may be due to the specific patient population or the limited field of view of both imaging techniques. Whole-body imaging modalities, such as positron emission tomography–computed tomography (PET-CT) or magnetic resonance imaging (MRI), may be more suitable for detecting distant metastases in patients with MPE [7,37].

This study assessed the accuracy of different US and CT findings in detecting MPE compared with histopathology. Both modalities demonstrated high specificity (100%) for several findings, including diaphragmatic pleural thickening, diaphragmatic pleural nodularity, pleural rind, chest wall invasion, and liver metastasis. This high level of specificity indicates that the presence of these findings on either US or CT is highly suggestive of malignancy [22].

However, the sensitivities and NPVs for most findings were relatively low, indicating that the absence of these features does not necessarily exclude the presence of MPE. The highest sensitivity was observed for diaphragmatic pleural nodularity in US (45.5%) and parietal pleural thickening and mediastinal thickening in CT (48.5%). These findings are consistent with previous studies that have reported moderate sensitivities for various US and CT findings in the diagnosis of MPE [38,39].

The PPVs for most US and CT findings were generally high, ranging from 85.7% to 100%, except for peripheral lung lesions (54.5% for US and 60% for CT). These high PPVs indicate that when specific findings are observed in either modality, there is a strong probability of malignancy. The logistic regression analysis in our study provided valuable insights into the relative importance of various imaging findings in predicting MPE. We found that diaphragmatic pleural nodularity detected in US emerged as the strongest predictor, with patients exhibiting this finding having approximately 9.5 times higher odds of MPE than those without nodularity. Similarly, the presence of a pleural rind in CT was strongly associated with MPE, conferring nearly seven times higher odds. These findings highlight the diagnostic usefulness of assessing diaphragmatic pleural involvement and the presence of a pleural rind, which is highly specific for malignancy. These results are consistent with previous studies that have also identified pleural nodularity and the presence of a pleural rind as strong predictors of MPE [22,32]. Notably, diaphragmatic pleural thickening in US and mediastinal thickening in CT were also significant predictors, but with slightly weaker associations. While certain findings, such as parietal pleural abnormalities, liver metastases, and chest wall invasion, showed modest associations with MPE, their predictive ability was limited compared with diaphragmatic and mediastinal findings. Logistic regression analysis allowed us to identify the most robust imaging predictors of malignancy, while accounting for potential confounding factors, thereby providing a more comprehensive understanding of the relative diagnostic value of different imaging characteristics.

Future research could explore the potential of advanced imaging techniques, such as contrast-enhanced US (CEUS) and diffusion-weighted magnetic resonance imaging (DW-MRI), in evaluating MPE. CEUS has shown promising results in distinguishing between benign and malignant pleural lesions based on their vascularity patterns [40]. On the other hand, DW-MRI has demonstrated potential in detecting and characterizing MPE, particularly when CT findings are inconclusive [41]. Furthermore, integrating imaging findings with other diagnostic modalities, such as thoracoscopy, pleural fluid analysis, and molecular biomarkers may further enhance the diagnostic accuracy and management of MPE [42].

This study had some limitations that should be considered when interpreting the results. First, it had a relatively small sample size of 54 patients, with 33 confirmed cases of MPE. Although valuable insights were obtained, a modest sample size may limit the generalizability of the findings. Future multicenter studies with larger patient cohorts are warranted to validate and build upon our results across diverse populations and clinical settings. Larger sample sizes would further strengthen the statistical power and reinforce the comparative evaluation of US and CT performance in predicting MPE. Second, the effect of operator experience on the diagnostic performance of US and CT was not assessed, which may have influenced the results in clinical practice. Third, this study did not evaluate the impact of imaging findings on patient management or outcomes, which would have provided additional context for the clinical relevance of the findings. Fourth, this study did not assess interobserver variability in the interpretation of the imaging findings, which could affect the reproducibility and reliability of the results. Fifth, we did not specifically analyze or compare the performance of US and CT in detecting septations and complex morphology within pleural effusions. Previous literature has suggested that lung US may have higher sensitivity than CT for revealing septated or loculated effusions, which can be an important indicator of malignancy [9]. By focusing primarily on evaluating pleural abnormalities, such as nodularity and thickening, we may have missed an opportunity to assess the complementary roles of these modalities in characterizing effusion complexity and internal architecture. Future studies should aim to incorporate a systematic evaluation of septated/complex effusions with US and CT in patients with suspected MPE.

## 5. Conclusions

In conclusion, this study showed that chest ultrasound and computed tomography have complementary roles in evaluating malignant pleural effusion. Ultrasound is particularly effective in detecting diaphragmatic pleural abnormalities, whereas CT is superior in identifying parietal pleural and mediastinal abnormalities. Both modalities had high specificity and relatively low sensitivity. By using a multimodal approach that combines the strengths of ultrasound and computed tomography, the diagnostic accuracy for suspected malignant pleural effusion can be optimized.

## Figures and Tables

**Table 1:** Patient Characteristics.

Variable	Value
Gender	
Male	34 (63)
Female	20 (37)
Age (years), Mean ± SD	50.8 ± 8.5
Pathological type of pleural effusions	
Malignant	33 (61.1)
Benign	21 (38.9)
Final diagnosis	
Benign pleural effusions	
Nonspecific inflammatory lesion	3 (5.6)
Tuberculosis	11 (20.4)
Pneumonia	7 (13)
Malignant pleural effusions	
Metastatic	21 (38.8)
Lung cancer	16 (29.6)
Adenocarcinoma	11 (20.4)
Small cell lung cancer	5 (9.2)
Breast cancer	4 (7.4)
Gastro-intestinal cancer	1 (1.9)
Mesothelioma	12 (22.2)

Unless otherwise indicated, the data are the number of patients with percentages in parentheses. SD, standard deviation.

**Table 2:** Chest US and CT Findings in 33 Patients with MPE.

Findings	US	CT	*p*-Value
Parietal pleural thickening			0.125
Absent	21 (63.6)	17 (51.5)	
Present	12 (36.4)	16 (48.5)	
Parietal pleural thickening			0.016
Absent	21 (63.6)	17 (51.5)	
<10 mm	3 (9.1)	3 (9.1)	
≥10 mm	9 (27.3)	13 (39.4)	
Parietal pleural nodularity			0.500
Absent	23 (69.7)	25 (57.8)	
Present	10 (30.3)	8 (24.2)	
Diaphragmatic pleural thickening			0.001
Absent	19 (57.6)	30 (90.9)	
Present	14 (42.4)	3 (9.1)	
Diaphragmatic pleural thickening			<0.001
Absent	19 (57.6)	30 (90.9)	
<10 mm	3 (9.1)	1 (3)	
≥10 mm	11 (33.3)	2 (6.1)	
Diaphragmatic pleural nodularity			<0.001
Absent	18 (54.5)	32 (97)	
Present	15 (45.5)	1 (3)	
Mediastinal thickening			0.002
Absent	27 (81.8)	17 (51.5)	
Present	6 (15.2)	16 (48.5)	
Pleural rind			<0.001
Absent	33 (100)	24 (72.7)	
Present	0 (0)	9 (27.3)	
Peripheral lung lesion			0.250
Absent	27 (81.8)	24 (72.7)	
Present	6 (18.2)	9 (27.3)	
Chest wall invasion			0.250
Absent	29 (87.9)	32 (97)	
Present	4 (12.1)	1 (3)	
Liver metastasis			0.500
Absent	30 (90.9)	32 (97)	
Present	3 (9.1)	1 (3)	

Data are presented as numbers of patients with percentages in parentheses. US, ultrasound; CT, computed tomography; MPE, malignant pleural effusion; p < 0.05, significant.

**Table 3:** Chest US and CT Findings in 21 Patients with Benign Pleural Effusion.

Findings	US	CT	*p*-Value
Parietal pleural thickening			1.000
Absent	19 (90.5)	19 (90.5)	
Present	2 (9.5)	2 (9.5)	
Parietal pleural thickening			1.000
Absent	19 (90.5)	19 (90.5)	
<10 mm	2 (9.5)	2 (9.5)	
>10 mm	0 (0)	0 (0)	
Parietal pleural nodularity			1.000
Absent	20 (95.2)	20 (95.2)	
Present	1 (4.8)	1 (4.8)	
Diaphragmatic pleural thickening			----
Absent	21 (100)	21 (100)	
Present	0 (0)	0 (0)	
Diaphragmatic pleural thickening			----
Absent	21 (100)	21 (100)	
<10 mm	0 (0)	0 (0)	
>10 mm	0 (0)	0 (0)	
Diaphragmatic pleural nodularity			----
Absent	21 (100)	21 (100)	
Present	0 (0)	0 (0)	
Mediastinal thickening			1.000
Absent	20 (95.2)	19 (90.5)	
Present	1 (4.8)	2 (9.5)	
Pleural rind			-----
Absent	21(100)	21(100)	
Present	0 (0)	0 (0)	
Peripheral lung lesion			1.000
Absent	16 (76.2)	15 (71.4)	
Present	5 (23.8)	6 (28.6)	

Data are presented as numbers of patients with percentages in parentheses. US, ultrasound; CT, computed tomography; *p* < 0.05, significant.

**Table 4:** Validity of Different US and CT Findings in Detecting MPE Using Histopathology as Reference Standard.

	Image	TP	FP	TN	FN	SN	SP	PPV	NPV
Parietal pleural thickening	US	12	2	19	21	36.4%	90.5%	85.7%	47.5%
	CT	16	2	19	17	48.5%	90.5%	88.9%	52.8%
Parietal pleural nodularity	US	10	1	20	23	30.3%	95.2%	90.9%	46.5%
	CT	8	1	20	25	24.2%	95.2%	88.9%	44.4%
Diaphragmatic pleural thickening	US	14	0	21	19	42.4%	100%	100%	52.5%
	CT	3	0	21	30	9.1%	100%	100%	41.2%
Diaphragmatic pleural nodularity	US	15	0	21	18	45.5%	100%	100%	53.8%
	CT	1	0	21	32	3%	100%	100%	39.6%
Mediastinal thickening	US	6	1	20	27	18.2%	95.2%	85.7%	42.6%
	CT	16	2	19	17	48.5%	90.5%	88.9%	52.8%
Pleural rind	US	0	0	21	33	0%	100%	0%	38.9%
	CT	9	0	21	24	27.3%	100%	100%	46.7%
Peripheral lung lesion	US	6	5	16	27	18.2%	76.2%	54.5%	37.2%
	CT	9	6	15	24	27.3%	71.4%	60%	38.5%
Chest wall invasion	US	4	0	21	29	12.1%	100%	100%	42%
	CT	1	0	21	32	3%	100%	100%	39.6%
Liver metastasis	US	3	0	21	30	9.1%	100%	100%	41.2%
	CT	1	0	21	32	3%	100%	100%	39.6%

US, ultrasound; CT, computed tomography; MPE, malignant pleural effusion; TP, true positive; FP, false positive; TN, true negative; FN, false negative; SN, sensitivity; SP, specificity; PPV, positive predictive value; NPV, negative predictive value.

**Table 5:** Logistic Regression Analysis for MPE.

Findings	US OR (95% CI)	*p*-Value	CT OR (95% CI)	*p*-Value
Parietal pleural thickening ≥ 10 mm	2.15 (1.02–4.53)	0.044	3.12 (1.28–7.62)	0.012
Parietal pleural nodularity	1.67 (0.79–3.55)	0.182	2.41 (0.92–6.31)	0.073
Diaphragmatic pleural thickening ≥ 10 mm	5.28 (2.19–12.72)	<0.001	2.67 (0.68–11.23)	0.157
Diaphragmatic pleural nodularity	9.45 (3.56–25.08)	<0.001	1.89 (0.22–16.01)	0.563
Mediastinal thickening	1.22 (0.49–3.04)	0.671	4.67 (1.83–11.92)	0.001
Pleural rind	----	----	6.82 (2.47–18.86)	<0.001
Peripheral lung lesion	0.72 (0.31–1.68)	0.448	1.14 (0.51–2.54)	0.748
Chest Wall Invasion	3.25 (0.98–10.76)	0.054	1.62 (0.19–13.73)	0.658
Liver Metastasis	3.89 (1.02–14.86)	0.047	1.78 (0.21–15.21)	0.603

US, ultrasound; CT, computed tomography; OR, odds ratio; CI, confidence interval; *p* < 0.05, significant.

## Data Availability

The clinical and imaging datasets used and/or analyzed during the current study are available from the corresponding author upon reasonable request.
